# Cardiotoxicity associated with anticancer therapies for gynecological tumors

**DOI:** 10.3389/fcvm.2026.1779990

**Published:** 2026-03-10

**Authors:** Liu Yang, Jixian Liao, Jing Li, Zanhong Wang

**Affiliations:** 1 Third Hospital of Shanxi Medical University, Taiyuan, China; 2 Shanxi Bethune Hospital, Taiyuan, China

**Keywords:** cardio-oncology, cardiovascular toxicity, gynecological cancers, management, monitoring, risk stratification

## Abstract

The continuous advancement in the management of gynecological cancers has contributed to improved patient survival. Nevertheless, cardiovascular toxicity resulting from anti-tumor treatments has emerged as a significant threat to long-term quality of life and non-cancer-related mortality. This review systematically elaborates on the cardiovascular risk of the conventional treatment of gynecological tumor viz chemotherapy, targeted therapy, immunotherapy, endocrine therapy and radiotherapy. The molecular mechanisms of each therapy will also be discussed, including oxidative stress, mitochondrial dysfunction, endothelial injury and immune-mediated inflammation. Additionally, we outline the major risk factors associated with anticancer therapy related cardiovascular toxicity and give an insight into monitoring, diagnosis and management of complications.

## 1 Introduction

Gynecological cancers are a major global health threat to women. The yearly incidence and mortality rates have increased significantly, with different epidemiology in various areas or population groups (1). The incidence of endometrial cancer in the US has been rising steadily over the last ten years. Furthermore, older adults are more likely to suffer from this type of cancer because of the diabetes and hypertension they suffer from, which complicates treatment and increases the chance of adverse effects (2, 3). In nations such as China, cervical and ovarian cancers continue to be fairly common, with a worrying shift toward younger age at diagnosis—this makes clinical management and long term follow up more complicated (4, 5). The present management of gynecological tumors has shifted towards a multidisciplinary approach incorporating surgical management, chemotherapy, radiotherapy, targeted therapies and immunotherapy, which has improved outcomes (6–8). Even though treatment strategies are getting refined continuously, long-term survival for gynecological cancer patients is still considered to be difficult to achieve, especially among the older adults, whose management is often more challenging (9, 10).

Due to advancements in anticancer therapies for gynecological tumors, the survival of patients is somewhat improved. However, adverse effects of certain anticancer therapies, particularly the cardiovascular toxicity, have become an important concern affecting quality of life and long-term outlook (11). Research studies have shown that 8.4% of patients receiving anthracyclines and trastuzumab develop cardiac toxicity. 4% of patients develop severe heart failure (12). The addition of chemotherapy to radiotherapy may put patients at a higher risk of cardiovascular complications, especially in the case of pelvic or thoracic targets. Coronary artery disease, myocardial fibrosis, valvular dysfunction and other conditions may result from this increased risk (13). The cardiovascular toxicity which is characterized by its variability and insidious onset and potential irreversibility is a leading cause of non-cancer-related deaths in cancer survivors. The mechanisms involved are complex, involving oxidative stress and apoptosis, endothelial dysfunction and other mechanisms, and are closely related to pre-existing cardiovascular health, type of drugs, total dosages and combinations of treatments (14). As such, an important challenge in gynecological oncology now is the systematic prevention and management of cardiovascular toxicity, alongside effective tumour control.

The purpose of this review encompassing the clinical manifestations, molecular mechanisms and risk factors associated with cardiovascular toxicity of treatments of gynecologic cancers. The article also summarizes the monitoring guidelines and preventive measures to regularize cardio-oncology in the management of gynecological tumors with a view to improving long-term quality of life in patients.

## 2 Treatment of gynecologic tumors associated with cardiovascular toxicity

Management of gynecological cancers is believed to have reached an integrated multidisciplinary level. Various treatment options, including chemotherapy and targeted treatment options, have greatly improved patient outcome but they have a significant cardiovascular risk (11) (Table 1). Research shows that nearly half (48%) of individuals will be affected by early cardiovascular complications during treatment, as well as 30% with long-term cardiac sequelae up to 13 years later (15). These toxic effects may affect a variety of cardiovascular components, including the myocardium and vascular system, and may lead to a range of clinical symptoms. Thus, health care providers must assess the cardiovascular toxicity of common treatment schemes to enable early detection and management.

**Table 1:** Cardiovascular toxicity profiles of common anticancer agents within gynecologic oncology.

Therapy category		Representative agents	Indication	Cardiovascular toxicities	Risk estimate
Chemotherapy	Anthracyclines	Doxorubicin pegylated liposomal doxorubicin(PLD)	Advanced or recurrent ovarian and uterine	Heart failure LV dysfunction	Doxorubicin: the decrease in LVEF >10%(50%): depends on drug dose PLD: HF(1.6%): Associated with high risk factors for CAD
	Alkylating Agents	Cyclophosphamide, Ifosfamide	Recurrent cervical and ovarian cancer	Myocarditis Pericarditis Arrhythmias Cardiogenic shock	Oral metronomic: rare high-dose: 7to28%
	Platinum-based	Cisplatin, Carboplatin	Gynecologic cancers	Thrombosis Arrhythmias	Cisplatin: venous thromboembolic event (11%) Carboplatin:rare
	Antimetabolites	5-Flurouracil/capecitabine	Cervix cancer Ovarian and endometrial cancers	Myocardial ischemia angina pectoris Acute coronary syndrome Heart failure	Rare
		Gemcitabine	Platinum-sensitive relapsed ovarian cancer	Coronary artery spasms Arrhythmias Heart failure	Rare
	Taxanes	Paclitaxel	Gynecologic cancers	Arrhythmia Hypotension	Arrhythmia: asymptomatic bradycardia (10%–20%)
		Docetaxel	Gynecologic cancers	—	Rare
		Bleomycin	Malignant ovarian germ cell tumors Malignant trophoblastic tumors	Hypotension Myocardial infarction Myocardial ischemia Coronary artery disease	Rare
		Vincristine	Ovarian and cervical cancers	Coronary artery spasm Myocardial infarction	Rare
Targeted Therapy	Anti-angiogenics	Bevacizumab, Pazopanib, Lenvatinib	Ovarian and cervical cancers	Hypertension Arterial and venous Thromboembolic Heart failure LV dysfunction	Bevacizumab: grade 3 to 4 hypertension(RR:19.01) thromboembolic(RR:4.99) Pazopanib: grade 3 to 4 hypertension(27%)
	PARP Inhibitors	Niraparib, Olaparib	Ovarian cancer	Hypertension Palpitations	Niraparib: hypertension (19%, grade 3 to 4 hypertension 9%) palpitation(10%)
	Anti-HER2	Trastuzumab	HER2-overexpressing uterine cancer Ovarian cancer	LV dysfunction Heart failure	Rare
Immunotherapy	Immune Checkpoint Inhibitors	Pembrolizumab	Endometrial cancers Cervical cancers	Myocarditis Pericarditis Arrhythmias	Overall incidence <1%
	Antibody-Drug Conjugates	Tisotumab vedotin,	Cervical cancers	—	—
		Mirvetuximab	Ovarian cancer	Hypertension	Grade 3 to 4 hypertension(0.14%)
Endocrine Therapy	SERMs	Tamoxifen	Ovarian cancer Endometrial cancers	Thrombotic Events Atherosclerosis Myocardial Infraction	—
	Aromatase Inhibitors	Letrozole, Anastrozole	Hormone-sensitive endometrial cancer Ovarian cancer	Hyperlipidemia	—
	GnRH Agonists	Leuprorelin	Endometrial cancers Ovarian cancer	Metabolic syndrome	—
	Progestins	Medroxyprogesterone	Endometrial cancers Ovarian cancer	Venous thromboembolic fluid retention	—

Rare: The frequency of toxicity <1% incidence in clinical trials or observational studies. Undefined frequency is represented by a dash (—).

CAD, cardiovascular disease; LV, left ventricular; LVEF, left ventricular ejection fraction.

### 2.1 Cardiovascular toxicity of chemotherapy drugs

Doxorubicin and Epirubicin are termed anthracycline agents. They are used as first or second line agents for the treatment of advanced or recurrent ovarian and endometrial cancer. These medications cause apoptosis of tumor cells by inserting themselves into the DNA double helix. They also induce permanent damage to the heart muscle (16). Some small studies involving patients with advanced epithelial ovarian malignancies indicated that almost 50% of patients suffered from cardiovascular toxicity as measured by left ventricular ejection fraction (LVEF) (11). Higher doses or with concurrent radiation can increase toxicity (17). Recent research shows that even subthreshold doses may cause subclinical cardiac injury that may occur years later (18). Pegylated liposomal doxorubicin (PLD) is a nanoformulation of doxorubicin, which modifies the distribution characteristics of the drug in the body reducing the incidence of cardiotoxicity of conventional formulations (19. Multiple studies have confirmed that the risk of clinically significant cardiotoxicity with PLD in gynecologic oncology patients is very rare. Cardiac adverse events remain uncommon even with long use or higher cumulative doses (19, 20). One study showed that all those who had heart failure due to the drug had significant cardiac risk factors before starting the drug (21). As a result, it is recommended in clinical practice to conduct cardiac function monitoring and long-term follow-up in high-risk population, namely older patients and patients with underlying heart disease during and after treatment.

Alkylating agents are a class of chemotherapeutic drugs that exert antitumor effects by inducing DNA strand breaks or cross-linking in cancer cells, with cyclophosphamide (CP) being one of their representative agents (22). In the history of ovarian cancer treatment, CP was once a first-line chemotherapy regimen but was gradually replaced by drugs such as paclitaxel (23). Traditional therapy often employed high-dose CP, accompanied by significant toxic side effects, with a cardiotoxicity incidence ranging from 7% to 28%. Clinical manifestations may include myocarditis, pericarditis, arrhythmias, and even cardiogenic shock (24). In recent years, CP has regained attention in the “metronomic chemotherapy” model, often playing an important role in improving the prognosis of advanced ovarian cancer patients and providing palliative care (25). Studies have demonstrated that oral metronomic CP (commonly administered at a daily dose of 50 mg) as monotherapy exhibits definitive activity and favorable tolerability in patients with recurrent, platinum-resistant, or refractory epithelial ovarian cancer (25, 26). However, potential synergistic cardiovascular toxicity may occur when CP is combined with other chemotherapeutic drugs, necessitating close monitoring of associated toxicity risks in clinical practice based on different combination regimens (22).

Cisplatin, carboplatin and other platinum-based agents exert their cytotoxic effects mainly by way of formation of DNA cross-links which occur both intra-strand and inter-strand. These cross-links impede replication and transcription. Platinum-based agents are the mainstay of first-line chemotherapeutic regimens for several gynecologic malignancies. The use of taxanes along with platinum-based agents is highly recommended for ovarian and cervical cancers. Heart muscle damage by platinum-based agents occurs mainly due to thrombosis (27). Chemicals that are made from platinum damage vascular endothelium and activate coagulation pathways, raising venous thromboembolism (VTE) risk (28). A cohort study on cancer, VTE event occurred in women with ovarian cancer in 11% within 12 months after the beginning of chemotherapy with cisplatin (29). Long-term use of cisplatin over time leads to increased atherosclerosis and coronary artery disease, especially in patients with risk factors for cardiovascular disease (CAD) (30). Additionally, cisplatin can cause slow heart rate that resolves on its own. Carboplatin is less cardiotoxic than other anti-cancer drugs, but it can prolong the QT interval. Increased risk of ventricular arrhythmia may occur, especially when used with other drugs that prolong the QT interval.

Antimetabolic agents block nucleic acid production in cancerous cells, hindering their growth and functioning as essential elements in chemotherapy for gynecological cancers. Notable examples include 5-fluorouracil (5-FU), capecitabine, and gemcitabine. In clinical practice, 5-FU is often used in cervix cancer concurrent chemoradiation and is first line or salvage therapy for gestational trophoblastic neoplasia (31, 32). The use of capecitabine is in the maintenance or recurrent settings of ovarian and endometrial cancers. Moreover, gemcitabine is an agent used in the case of platinum-sensitive relapsed ovarian cancer (33). While there is not much literature available on gynecological malignancies, the rates of cardiotoxicity observed with 5-FU and its prodrug capecitabine for solid organ tumors range from 1% to 19% (34, 35). Myocardial Ischemia, angina pectoris, and chest pain are the most common clinical manifestations. They may be associated with electrocardiogram (ECG) changes like ST segment or T-wave changes. In serious cases, it may cause acute coronary syndrome, heart failure, ventricular arrhythmias or sudden cardiac death (36). A meta-analysis suggests that patient-related potential risk factors for fluoropyrimidine-related cardiotoxicity include age ≥65 years, history of coronary arterial disease, concurrent radiotherapy, hypertension, hypercholesterolemia, diabetes mellitus, high BMI, smoking. High drug doses and continuous infusions also add to cardiotoxicity (37). Moreover, recurrence of cardiotoxicity can occur in about 82%–100% of 5-FU patients after re-exposure to 5-FU (38). Gemcitabine-related cardiotoxicity is relatively uncommon. Recent clinical applications have recorded an increasing number of arrhythmia cases, with certain patients experiencing coronary artery spasms, which may worsen to heart failure or dilated cardiomyopathy in serious cases (39–41). Plus, gemcitabine might lead to capillary leak syndrome, which can worsen into cardiogenic shock in serious cases especially when combined with either doxorubicin or paclitaxel (42).

Taxanes, including paclitaxel and docetaxel, are essential chemotherapy drugs used to treat gynecological malignancies through inhibition of microtubule depolymerization (43). The main cardiovascular side effects of these agents are arrhythmias and hypotension. While receiving paclitaxel, around 10%–20% of people may develop a slow heart rhythm. This is mostly asymptomatic. Furthermore, stopping the drug is often enough for its resolution. A small subset of patients, particularly those with pre-existing conduction system disease or simultaneous exposure to other cardiotoxic medications, may experience severe arrhythmias and myocardial injury (44, 45). The rate of hypotension is 5%–10% and is generally related to an excessively infusion rate. It may also occur due to the peripheral vasodilation caused by released histamine. In severe cases, this may necessitate stopping the infusion temporarily and providing supportive treatment (43). Although docetaxel and paclitaxel exhibit comparable antitumor efficacy, their toxicity profiles differ substantially. Clinical trial of carboplatin-paclitaxel vs. carboplatin-docetaxel. Comparing docetaxel-carboplatin vs. paclitaxel-carboplatin in patients with ovarian cancer demonstrated; that docetaxel is associated with a higher hematological toxicity but a lower cardiac toxicity (46).

Bleomycin is mainly used in gynecologic oncology as a first-line standard chemotherapeutic agent for malignant ovarian germ cell tumors and also used for malignant trophoblastic tumors (47). It works by causing the DNA strands to break and thus prevents the multiplication of tumor cells. While bleomycin exhibits no known cardiotoxic effects, in rare instances, it may trigger hypotension, myocardial infarction, myocardial ischemia, or coronary artery disease (48). Through direct damage to vascular endothelial cells, oxidative stress and inflammatory mechanisms could be responsible for the mechanisms involved.

Vincristine, an agent that inhibits microtubule formation, is sourced from a plant that prevents tubulin polymerization and produces the arrest of spindle-fibers. As a result, the mitosis of cancer cells is inhibited at the metaphase stage (49). In gynecologic oncology, it mainly acts as a synergistic adjuvant in combination therapy. Its cardiovascular toxicity is relatively rare, with very few cases reported of coronary artery spasm or myocardial infarction (50, 51). Because of the infrequency of its use as monotherapy, it is difficult to define the cardiac toxicity risk of this agent (52).

### 2.2 Cardiovascular toxicity of targeted therapy drugs

Owing to advances in molecular biology, targeted therapy has now become an important strategy for gynaecological cancers. This method selectively inhibits the signaling pathways used by tumor cells to proliferate, metastasize and survive, leading to better outcomes for patients (53, 54). Nonetheless, anti-cancer therapies targeting tumor cells can damage the cardiovascular system. In particular, angiogenesis, cell growth as well as immune system all seem to have overlapping signalling pathways in tumor as well as the cardiovascular system (55). Common targeted agents used to treat gynecological tumours are classified by targets and mechanisms into anti-angiogenics therapies, PARP inhibitors, anti-HER2 agents and small molecule tyrosine kinase inhibitors (TKIs) (53).

Anti-angiogenic therapies attack tumors by preventing the vascular endothelial growth factor (VEGF) signal and its receptors, thus shutting down tumor angiogenesis (56). These agents are recommended in both frontline and recurrent settings of ovarian and cervical cancers. he important example is the monoclonal antibody bevacizumab, and a small-molecule VEGFR tyrosine kinase inhibitor (VEGFR-TKI) is pazopanib (57). Cardiovascular adverse events associated with these medications encompass hypertension, proteinuria, thromboembolic complications, left ventricular dysfunction, and myocardial ischemia (58). Bevacizumab is a humanized anti-VEGF monoclonal antibody used for the treatment of ovarian and cervical cancers, which has significant cardiovascular morbidity, including hypertension, arterial and venous thromboembolic, and heart failure (59). A meta-analysis of a phase III clinical trial involving patients with recurrent ovarian disease treated with bevacizumab indicated a high risk of grade 3 to 4 hypertension (RR 19.01, 95% CI: 7.77–46.55; *p* < 0.00001) and arterial thromboembolic events (ATE) (RR 4.99, 95% CI 1.29–19.27; *p* = 0.02) (60). VEGFR-TKIs cardiovascular adverse reactions akin to those of bevacizumab, including hypertension, cardiomyopathy, left ventricular dysfunction, heart failure, QT prolongation, and myocardial ischemia (58). For instance, a study investigating pazopanib maintenance therapy for advanced epithelial ovarian cancer revealed that 27% of patients receiving pazopanib experienced grade 3 or higher hypertension (61). Lenvatinib and pembrolizumab have been approved for combination therapy in endometrial cancer (62). Summarized studies indicate that, in the treatment of recurrent endometrial cancer with lenvatinib and pembrolizumab, 41.95% of patients developed hypertension, with 21.49% experiencing grade 3 or 4 hypertension (63). The occurrence of heart failure or cardiomyopathy has been reported for some of the VEGFR-TKIs, including lenvatinib and sunitinib.

PARP inhibitors block the repair of DNA damage in tumor cells. They work best in patients with ovarian and breast cancer with a BRCA mutation or homologous recombination deficiency. Thus, they are vital agents in gynaecological cancer therapy (64). The most common PARP inhibitors are olaparib, rucaparib, niraparib, and Fuzuloparib. The cardiovascular toxicities mainly present themselves as hypertension and palpitations, niraparib being the most relevant (65, 66). According to the NOVA study, 19% of individuals developed hypertension, with grade Ⅲ-IV hypertension having an incidence of 9%, as well as 10% of individuals had palpitations (67). Niraparib may lead to severe hypertension, hypertensive crises which occur infrequently, that happens specially in the first month of treatment (68).

Anti-HER2 therapy exerts antitumor effects by blocking the HER2 signaling pathway, such as with the monoclonal antibody trastuzumab (69). This treatment is being indicated for HER2-overexpressing uterine cancer, a phenotype responsible for about 30% of uterine serous carcinoma (70). It mainly causes left ventricular dysfunction and heart failure in the heart. Unlike anthracycline-induced myocardial injury, which is typically irreversible, trastuzumab-associated cardiotoxicity is generally reversible. After discontinuation of the drug, approximately 70%–80% of patients experience recovery of LVEF (71). It should be noted that this toxicity is not exhibit a clear dose-dependent, can occur early on in treatment or several months after stopping treatment, and some patients are asymptomatic (72).

Thus, cardiac function must be actively monitored throughout the treatment. Currently, anti-HER2 therapy does not seem to be signidicantly cardiotoxic in treating uterine serous carcinoma (73). The majority of existing evidence-based data in this area emerges from breast cancer populations. Consequently, risk assessment for gynecologic oncology patients often relies on reasonable extrapolation of this evidence.

Furthermore, the use of multi-target tyrosine kinase inhibitors (e.g., imatinib) and mTOR inhibitors (e.g., everolimus) in specific gynecological cancer subtypes, is on the rise (74, 75). Despite the low overall rate of cardiovascular adverse events linked to these drugs, monitoring should be conducted thoughtfully. Chronic myeloid leukaemia and gastrointestinal stromal tumours are the primary indications for imatinib. Its use in gynaecological oncology, particularly in ovarian cancer, is preclinical or early exploratory (74). Clinical trials on a larger scale are needed to confirm the efficacy and safety of Imatinib. Drug-related cardiac adverse reactions such as heart failure have been reported infrequently in patients with gastrointestinal stromal tumours, approximately 0.5%–1.7% (76). Patients who are 65 years or older, or have underlying heart disease are at higher risk. Everolimus, a second-line treatment for metastatic endometrial cancer, poses a cardiovascular risk, mainly maladies of lipoprotein metabolism, can lead to hyperlipidemia. In a randomized Phase II trial of (*n* = 37), hypertriglyceridemia developed in up to 54% of everolimus treated patients (77).

### 2.3 Immunotherapy drugs

The immune system fights against cancer cells with the help of immunotherapy. It enhances the body's anti-tumor response. Various various malignant tumors, immunotherapy is shaping up to be an effective strategy (78). In gynecological oncology, immune checkpoint inhibitors (ICIs) and antibody-drug conjugates (ADCs) are increasingly being applied (79). ICIs enhance T cell anti-tumor activity by blocking immunosuppressive pathways such as CTLA-4 and PD-1/PD-L1. Currently, pembrolizumab is approved for second-line treatment of endometrial and cervical cancers with high microsatellite instability or mismatch repair deficiency (80). Although cardiovascular immune-related adverse associated with ICIs are rare (overall incidence <1%), they carry high mortality risk (81) These events mainly include myocarditis, pericarditis, arrhythmias, and vasculitis, with myocarditis mortality rates ranging from 25% to 50% (82).

ADCs consist of monoclonal antibodies conjugated to cytotoxic payloads, which target tumor-specific antigens to deliver therapeutic toxins. Notable advancements have been made in ADC application for gynecological cancers (83). For example, tisotumab vedotin (targeting TF antigen) is used for recurrent/metastatic cervical cancer, while mirvetuximab soravtansine (MIRV, targeting FR*α*) treats platinum-resistant ovarian cancer (84). Regarding cardiovascular safety, current data indicate that the risks associated with these two drugs are manageable (85). The main dose-limiting toxicities associated with tisotumab vedotin treatment are mainly ocular events, bleeding, and peripheral neuropathy, with infrequent occurrences of clinically significant cardiovascular toxicity (86). According to a clinical data, MRIV delivers an acceptable safety profile in patients with ovarian cancer with hypertension at a rate of around 0.14% among grade ≥3 adverse events (87). Overall, ADCs have a good risk-benefit profile in gynecological cancers. Further larger studies are still needed to determine the long-term cardiovascular safety and durable efficacy of these agents along with their most suitable clinical application.

Chimeric antigen receptor T-cell (CAR-T) therapy, which involves the genetic engineering of a patient's own T-cells to express chimeric antigen receptors that target specific tumor antigens, represents a significant advancement in the field of oncology. However, its application in gynecologic oncology remains largely exploratory (88). Notably, the associated safety risks merit careful consideration; systemic inflammatory responses, such as cytokine release syndrome, can lead to hemodynamic instability and cardiovascular complications. Although clinical data specific to gynecological malignancies are limited, insights gained from other tumor types suggest that future clinical applications should integrate cardiovascular monitoring into the safety management protocol (89).

### 2.4 Endocrine therapy

Endocrine therapy is administered for the treatment of hormone receptor-positive endometrial cancer and ovarian granulosa cell tumours. Using the therapy results in a reduction in the estrogen level of the patient or blocks the estrogen receptors of the patient (90). Key drugs are selective estrogen receptor modulators such as tamoxifen, aromatase inhibitors (AIs) such as letrozole and anastrozole, gonadotropin-releasing hormone (GnRH) agonists such as leuprorelin, and progestins such as medroxyprogesterone and megestrol. According to the study, the effect of these treatments on the body's hormone balance is complicated. Likewise, these treatments have a complex effect on cardiovascular health complication. Also, prolonged use of these treatments can cause an increase in the risk of hypertension, thrombosis, coronary artery disease and heart failure (91).

Tamoxifen, a selective estrogen receptor modulator, is an essential drug in the treatment of gynecological tumors with dual effects on the cardiovascular system (90). Tamoxifen has estrogen agonist effects on cardiovascular tissues, which may improve lipid profile and lower coronary heart disease risk in postmenopausal patients. However, it does raise the risk of VET due to its estrogenic effects (92). AIs work by blocking the aromatase enzyme to lessen the production of estrogen in peripheral tissues. Further, AIs are now offered as adjuvant treatment for hormone-sensitive endometrial cancer (77). According to large-scale meta-analyzes, AIs offer substantial safety benefits over tamoxifen, reducing the risk of venous thromboembolic events by approximately 47% (93). Leuprorelin and Goserelin are gonadotropin-releasing hormone agonists. They reduce estrogen levels by suppressing pituitary gonadotropin secretion. These medicines are mainly used for the treatment of ovarian cancer associated with endometriosis and recurrent hormone-sensitive gynaecological tumours. Long term GnRH suppression can cause a range of metabolic disorders, including insulin resistance, dyslipidemia, and obesity, with an increased risk of cardiovascular events including hypertension, myocardial infarction and stroke (94). Progestins, including medroxyprogesterone acetate and megestrol acetate, are useful in conservative or palliative treatment of endometrial cancer with cardiovascular risks include thromboembolism and fluid retention (95).

### 2.5 Radiotherapy

Radiotherapy plays an important role in definitive and postoperative adjuvant treatment of gynaecological malignancies, like cervical cancer and endometrial cancer (96). However, due to increment in the number of patients surviving, the radiation therapy long-term damaging effects on the heart are growing (97). Large cohort studies have identified radiotherapy as an independent risk factor for decreased cardiovascular-specific survival in patients with cervical and corpus (endometrial) cancers (98). The studies suggest the reason could be inflammatory activation, production of reactive oxygen species and oxidative stress which act in radiotherapy-induced CAD pathogenesis. Contrary to breast cancer radiotherapy that directly hits the thorax, conventional pelvic radiotherapy for gynecological cancers is given remote from the heart. This drastically lessens risks like radiation-induced heart issues, plus artery and valve problem (97).

Research indicates that the doses of radiation received by breast cancer patients are correlated with accelerated coronary artery calcification and an increased risk of coronary heart disease in the long-term (99). In some patients with locally advanced cervical cancer, the situation is different and extended field radiotherapy is necessary to include the para-aortic lymph nodes. The inferior border of the heart may lie within the radiation field in these cases or receive scattered doses. The frequency of disease like heart attack indeed appears higher in cervical cancer patients on radiotherapy, reports studies (100). For these situations, the heart should be defined as an organ-at-risk in radiotherapy treatment planning. Radiation treatment should use advanced techniques such as intensity-modulated radiation therapy (IMRT) to provide a sufficient dose to the target while ensuring that the dose to the heart, in particular, the apex of the heart is “as low as reasonably achievable” (96).

## 3 Mechanisms of cardiovascular toxicity induced by anticancer therapies

**Figure 1:**
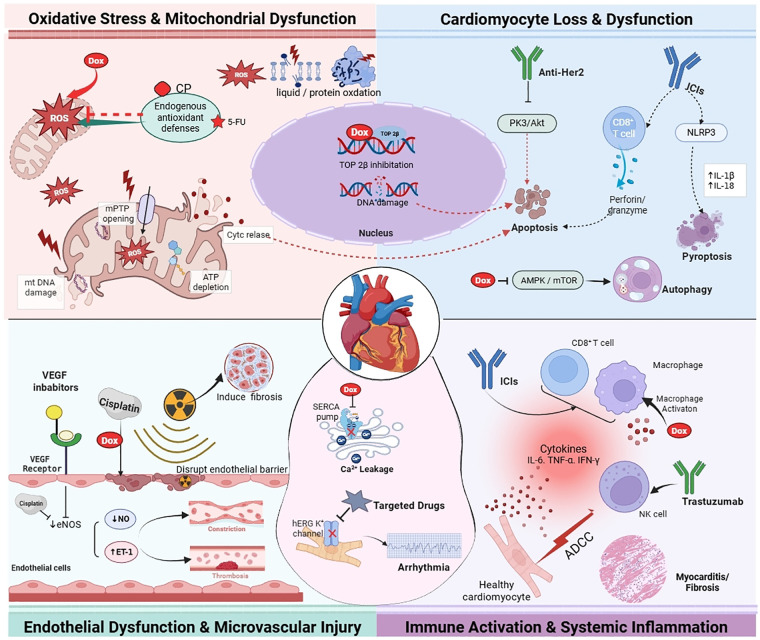
A multi-mechanism schematic diagram of cardiovascular toxicity related to anti-tumor therapy. The diagram shows the key pathways leading to cardiac injury caused by different anti-cancer drugs from four aspects: (1) Oxidative stress and mitochondrial dysfunction; (2) Loss and dysfunction of cardiomyocytes; (3) Endothelial dysfunction; (4) Immune inflammatory response. Overall, it reflects that chemotherapy, targeted therapy and immunotherapy jointly promote the occurrence and progression of cardiovascular toxicity through multiple pathways such as oxidative stress, cell death, endothelial injury and immune inflammation. DOX: doxorubicin; CP: cyclophosphamide; 5-FU: 5-fluorouracil; ICIs: immune checkpoint inhibitors; ROS: reactive oxygen species; mtDNA: mitochondrial DNA mPTP: mitochondrial permeability transition pore; ATP: adenosine triphosphate; cyt c: cytochrome c; Top2*β*: topoisomerase 2*β*; eNOS: endothelial nitric oxide synthase; NO: nitric oxide; ET-1: endothelin-1; SERCA: sarco endoplasmic reticulum Ca2+-ATPase; ADCC: antibody-dependent cell-mediated cytotoxicity. Created in BioRender (174).

Cardiovascular injury due to anticancer therapies is a complex process that occurs through multiple stages and mechanisms. It initiates a cascade of events from molecular and cellular level to organ dysfunction (Figure 1). To develop effective prevention and management strategies in this field, it is important to have a thorough understanding of the underlying mechanisms as well as an accurate assessment of risks.

### 3.1 Oxidative stress and mitochondrial dysfunction

The main cause of heart damage caused by anthracyclines is oxidative stress (101). Within cardiac mitochondria, the quinone part of anthracyclines undergoes single-electron reduction resulting in reactive oxygen species (ROS). Lipid peroxidation, protein denaturation and DNA injury are triggered under these conditions. This together with the inhibition of TOP2B affects genomic integrity and triggers apoptotic pathways. Moreover, activation of NADPH oxidase (NOX) and depletion of endogenous antioxidants further enhance the cycle of oxidative stress and inflammation (102, 103). Mitochondria are critical for the production of ROS, which refers to reactive oxygen species. Dysfunction in mitochondria is due to the decreased efficiency of the electron transport chain. Moreover, there is impaired ATP synthesis due to this dysfunction. Similarly, there is altered mitochondrial dynamics due to their dysfunction. Ultimately, all this results in the failure of cardiac energy metabolism (104). Several other agents induce different pathways of oxidative damage: the metabolites of CP deplete glutathione (105); 5-FU hinders the clearance of ROS by inhibiting dihydropyrimidine dehydrogenase (106); and anti-angiogenic agents impair nitric oxide synthesis by inhibiting endothelial nitric oxide synthase (eNOS), resulting in the disruption of redox homeostasis (58) whilst ICIs also induce oxidative damage via the over-activation of immune cells (107, 108).

### 3.2 Cardiomyocyte loss and dysfunction

Over the course of anticancer treatment, the death of cardiac muscle cells, or cardiomyocytes, is a critical factor in the development of permanent cardiac dysfunction. It happens through a process called apoptosis, in addition to autophagy and pyroptosis. Anthracyclines activate p53 through TOP2B inhibition, causing mitochondrial permeability transition pore opening and caspase-dependent apoptosis (12). Targeted therapies like trastuzumab are able to stop the function of HER2 as well as its downstream PI3 K/Akt survival pathway restore the balance between pro-apoptotic and anti-apoptotic proteins and induce cardiomyocyte apoptosis (109). ICIs cause cardiomyocyte death using perforin/granzyme and cross-reactive immune responses (perforin/granzyme) (107). Other than apoptosis, altered autophagy and pyroptosis may also contribute to cardiovascular harm. Inhibition of the AMPK/mTOR pathway promotes excessive autophagy at the level of cardiomyocytes ultimately damaging organelles and depleting energy to injure the heart (110). ICIs activate the NLRP3 inflammasome that induces pyroptosis mainly involving the release of IL-1β/IL-18 and rupture of the cell membrane that finally damages the heart (111).

### 3.3 Endothelial dysfunction and microvascular injury

The vascular endothelium is a major functional target of anticancer therapy-induced cardiovascular toxicity. Its dysfunction is responsible for impairment of microvascular integrity and coagulation homeostasis disturbance with vascular dysfunction (112). Chemotherapy agents like anthracyclines and platinum compounds can directly damage endothelial cells. Endothelial cell damage is associated with reduced capillary density, also termed microvascular rarefaction. Microvascular rarefaction after chemotherapy can ultimately lead to myocardial hypoperfusion as well as tissue edema (104). Cisplatin is an alkylating agent that inhibits eNOS phosphorylation. This inhibition causes a decrease in NO production. It can also enhance expression of intercellular adhesion molecule 1. This will lead to vasospasm and hypertension (27).

Anti-angiogenic agents (e.g., VEGF inhibitors) interfere with the VEGF/VEGFR signaling cascade, diminishing eNOS activity and thus reducing the vasodilator NO while increasing the vasoconstrictor endothelin-1 contributing to hypertension and endothelial-dependent vasodilation impairment (58). In addition, VEGF inhibitors affect the vascular wall integrity. Their use increases the risk of arterial thrombosis, possibly because they increase platelet activation and Tissue factor pathway inhibitor activity (113).

Moreover, ICI-induced immune vasculitis recreates endothelial barrier damage and causes a procoagulant microenvironment (114). Radiotherapy accelerates atherosclerosis in large vessels in cancer patients receiving anticancer therapy. In addition, it causes microvascular occlusive lesions due to effects of ionizing radiation and sustained activation of profibrotic factors like TGF-*β* (115).

### 3.4 Immune activation and systemic inflammation

Immune activation and inflammatory response are the likely causes of cardiovascular toxicity caused by ICIs, targeted therapies and conventional chemotherapy. Immune cell infiltration, excessive cytokine release and autoantibody formation characterize these processes. ICIs can induce autoimmune myocarditis by affecting the CTLA-4/PD-1-PD-L1 pathway in T cells. This leads to a pathological infiltration of CD8+ T cells and macrophages into myocardial interstitium with high pro-inflammatory cytokine levels (IL-6, TNF-α, IFN-*γ*) (116). Conventional chemotherapeutic agents (namely anthracyclines) activate TNF receptors via the release of TNF-α, which triggers macrophages and monocytes. Moreover, through the pathway of mitogen-activated protein kinases, these drugs modify inflammatory cytokines (112). Studies show that serum IL-6 levels may increase in patients receiving anthracyclines, and this increase is independently associated with left ventricular dysfunction (117). Drugs that inhibit angiogenesis compromise the integrity of vascular endothelial cells by functioning through the inhibition of the VEGF/VEGFR pathway and enhancement of chemokines that include IL-6, TNF-α etc. Thus, the extravasation of circulating inflammatory cells into myocardial tissues aggravates the “inflammation-fibrosis” cycle (118). Furthermore, trastuzumab is the HER2 inhibitor which even studies have shown to have direct cytotoxic effects on cardiomyocytes by activating antibody-dependent cell-mediated cytotoxicity through natural killer cells (119).

Inflammatory responses can cause problems such as heart cell death, scarring, and electrical issues. IL-1β directly inhibits the expression of myocardial contractile proteins, including the myosin heavy chains, while TNF-α increases the expression of matrix metalloproteinases via activation of NF*κ*B, leading to extracellular matrix degradation and hypertrophy of the ventricle (120). Also, due to chronic inflammation, fibroblasts may differentiate into myofibroblasts that may lead to the secretion of collagen types I and III that may increase myocardial stiffness. The above mentioned process is more pronounced in radiotherapy patients because that can enhance the fibrotic signal through TGF-*β* induction (121).

### 3.5 Others

Apart from the mechanisms mentioned above, anticancer therapies can also induce cardiotoxicity by disrupting calcium homeostasis, influencing epigenetic regulators, and altering neuroendocrine systems. Anthracyclines, through the inhibition of sarcoplasmic reticulum Ca2^+^-ATPase and ryanodine receptor, cause transient dysregulation and aberrant accumulation of intracellular calcium in cardiomyocytes, ultimately leading to decreased myocardial contractility and increased arrhythmogenicity (120) therapies like the multi-target tyrosine kinase inhibitors (e.g., Sorafenib) may block the hERG K^+^ channel, thus prolonging cardiomyocyte action potential duration, which may induce TdP. This risk is strongly dose-dependent and influenced by other drugs, e.g., macrolide antibiotics (122, 123). Epigenetic regulation plays a critical role in persistent cardiotoxic effects. Anthracyclines may activate DNA methyltransferase or inhibit histone deacetylase, resulting in the silencing of cardioprotective genes (e.g., FOXO3a, SIRT1), leading to epigenetic changes that may last for years and increase the likelihood of late-onset cardiomyopathy (124).

There is a neuroendocrine dysregulation in anti-angiogenic therapy-related hypertension. VEGF working inhibitors are utilized to reduce sodium reabsorption in the renal proximal tubule and activate the renin-angiotensin-aldosterone system. These events increase extracellular fluid volume and elevate peripheral vascular resistance. These effects may be mediated by the sympathetic nervous system (125, 126). Additionally, the immune therapies can impair the functioning of the thyroid causing destructive thyroiditis and may indirectly cause tachycardia or other cardiovascular events (127). A thorough assessment of the endocrine-cardiac axis is important.

## 4 Risk factors associated with cardiovascular toxicities

The risk of cardiovascular toxicity varies from person to person and is affected by patient characteristics, tumor type, treatment, and genetics.

### 4.1 Patient intrinsic factors

Age is an essential predictor of cardiovascular toxicity because of myocardial cell aging. In patients with gynecologic malignancies, certain baseline characteristics have been significantly associated with the risk of cardiovascular toxicity of antineoplastic drugs. Old age is an important risk factor mainly due to the reduced ability of the heart to compensate because of an ageing of cardiomyocytes and an increase in comorbidities (128). The menopausal status, especially surgically induced early menopause from bilateral oophorectomy, acts as an independent risk predictor for acute cardiac events (129). A prior history of CAD, including high blood pressure, coronary heart disease, and cardiac insufficiency, is a strong basis for toxicity development, with high blood pressure being the most common risk factor (130). Hypertension reduces heart endurance due to cardiac hypertrophy and fibrosis. According to research, patients with baseline hypertension have an elevated risk of developing grade 3–4 hypertension when treated with VEGFi (118). In addition, some unhealthy lifestyle factors, including smoking, alcohol abuse, obesity, and physical inactivity, increase the risk of cardiovascular toxicity by increasing oxidative stress and other metabolic disorders (128).

### 4.2 Tumor-specific factors in gynecological cancers

The likelihood of cardiovascular adverse events is related to the biological characteristics of specific tumor types and the intensity of the necessary treatments (131).

Different cancer types have unique risk levels. This is due to treatment protocols as well as the metabolic status of patients. The first-line treatment for patients with ovarian cancer consists of platinum-based chemotherapy combined with paclitaxel. Compared with cervical cancer treatment, this regimen is likely associated with higher rates of vascular complications, including hypertension and thrombosis. Patients with endometrial cancer may present with metabolic disorders like obesity and diabetes and have an inherent cardiological high-risk background (132). Moreover, the commonly used conservative progestin treatment may worsen fluid retention and thrombotic risk. The core factor determining treatment intensity and consequently cardiovascular risk is tumor staging. In cases of advanced-stage (III-IV) disease, patients potentially require longer cycles of chemotherapy (6–8 cycles per patient), which results in greater cumulative doses of drug and significantly higher cardiovascular toxicity risk (133). The sequential use of PARP inhibitors, immunotherapy and other agents for recurrent tumors causes significant cumulative toxicity. For instance, the combination of trastuzumab with anthracyclines raises cardiomyopathy risk to about 7 fold (134). Some intensive treatment techniques may also challenge cardiac function. The use of hyperthermic intraperitoneal chemotherapy, to enhance local control rates in ovarian cancer, with an operating temperature of 42–43 °C, may theoretically increase metabolic demand and oxidative stress in the myocardium. Nonetheless, its exact correlation with cardiovascular toxicity still needs to be clarified by prospective studies (135).

### 4.3 Treatment-related factors

Drug dosage, category, and whether multiple drugs are used in combination are key risk factors leading to cardiovascular toxicity. The danger of anthracyclines demonstrates a dose-response manner (an increase in cardiovascular damage happens with a 300 mg/m^2^ cumulative dose of doxorubicin) (136). Different drug classes produce different toxicities. For instance, the major toxicities of anti-angiogenic agents like bevacizumab include hypertension and thrombosis, while the risk of experiencing hypertension increases with dose (118). HER2-targeted therapies have left ventricular dysfunction as the major toxicity. The ICIs have different toxicity profiles depending on the drug. CTLA-4 inhibitors are more likely to cause myocarditis than PD-1 inhibitors. Moreover, dual combination immunotherapy poses a greater risk than monotherapy (111, 137, 138). Furthermore, the frequency of ICI administration may also influence toxicity. For example, a biweekly schedule rather than a triweekly schedule may heighten inflammation-related toxicity due to prolonged immune activation. Combination treatment strategies can result in additive or synergistic toxicity. For example, the sequential or concurrent use of anthracyclines and trastuzumab has been shown to markedly increase the risk of heart failure (72). Concurrent chemoradiotherapy also presents higher cardiovascular risks compared with single modality treatment (121).

### 4.4 Genetics and molecular markers

Treatment inducement cardiac injury sees considerable interindividual variability, which are also associated with genetic factors (139). Genetic differences in drug-metabolizing enzymes, including carbonyl reductases CBR1 and CBR3, uridine diphosphate glucuronosyltransferase UGT1A6, and sulfotransferase SULT2B1, can modify the activation and detoxification processes of anthracyclines and other drugs, as well as their clearance, thus modifying exposure and cardiotoxicity (140).

Patients with germline mutations in BRCA1 or BRCA2 may exhibit impaired DNA repair capacity in cardiomyocytes (141). Studies have shown that the LVEF decline after anthracycline-based chemotherapy in these patients is significantly greater than non-carriers demonstrating increased vulnerability of heart. Based on genome-wide association studies, scientists have identified several predictive genetic markers. The H63D polymorphism in the HFE gene has been validated as an independent risk factor for subclinical cardiac injury, which can assist in identifying patients at high risk for early intervention (142). In another study, it was found that single nucleotide polymorphism rs2229774 in retinoic acid receptor gamma gene (RARG) is significantly correlated with an increase in risk of anthracycline-induced cardiotoxicity (143). A deeper understanding of these genetic mechanisms provides novel targets for developing precision cardioprotective strategies. The use of RARG agonists such as CD1530 has shown promise in delivering cardioprotective effects in preclinical models for carriers of the RARG rs2229774 risk allele (143).

## 5 Monitoring, diagnosis, and evaluation of cardiovascular toxicity

### 5.1 Common detection techniques

Modern cardio-oncology depends on the integrated application of various diagnostic techniques to facilitate the early identification and accurate assessment of cardiovascular toxicity.Throughout the anti-tumour treatment, clinicians should dynamically use cardiac biomarkers, imaging and other approaches to identify cardiovascular toxicity early. Discovering impaired cardiac function before the appearance of obvious clinical signs entails the option for early intervention. This content introduces the assessment indicators of cardiovascular toxicity from three aspects: Imaging Monitoring, Biomarker Monitoring, and Functional Studies and Clinical Evaluation.

#### 5.1.1 Imaging monitoring

Echocardiography is the primary imaging modality to monitor therapy-related cardiotoxicity in cancer. Standard echocardiography provides a comprehensive assessment of cardiac anatomy, including left ventricular dimensions, wall thickness, and valve structure, while also evaluating global cardiac function through parameters such as LVEF and diastolic function. The LVEF, which is often relied on for routine monitoring, has limited sensitivity as its decline often means serious damage to the myocardium. In contrast, speckle-tracking echocardiography allows for the early detection of subclinical left ventricular dysfunction by quantitatively evaluating myocardial strain in the longitudinal, radial, and circumferential directions. Significantly, global longitudinal strain (GLS) has become the commonly used most sensitive parameter in clinical practices. Studies have shown that in patients with gynecologic cancers, GLS declines abnormally before LVEF reduction, indicating myocardial injury early on, even when LVEF remains within normal limits (144). Furthermore, cardiac magnetic resonance (CMR) is often viewed as the “gold standard” for assessing cardiac structure and function non-invasively (144). The use of tissue tracking technology in CMR allows for a quantitative analysis of myocardial deformation with precision.

#### 5.1.2 Biomarker monitoring

Cardiac troponin is a biomarker specific to myocardial cell injury. There are two types of cardiac troponin: cardiac troponin T and cardiac troponin I. Troponin levels increase when myocardial cells undergo necrosis or apoptosis. The advancement of high-sensitivity cardiac troponin (hs-cTnT/I) detection technologies has resulted in the creation of assays capable of detecting very small amounts of myocardial damage that standard techniques may overlook (145). Serial monitoring of hs-cTnT could help detect myocardial injury early on during anthracycline or trastuzumab therapy, predicting left-ventricular dysfunction.

The main source of natriuretic peptide, especially BNP and NT-proBNP, are ventricular myocytes secreting them upon volume and/or pressure overload. These peptide biomarkers are important to measure the ventricular wall stress and cardiac dysfunction (145, 146). High levels of these peptides are strongly correlated with the presence and severity of heart failure. Combined monitoring of hs-cTnT and NT-proBNP facilitates the identification of subclinical cardiac injury before clinical symptoms appear, enabling timely adjustments or initiation of heart failure therapy.

Emerging markers are increasingly being incorporated into clinical practice, in addition to known biomarkers (147). Research is under way to include C-reactive protein, myeloperoxidase, and galectin-3 in a multi-marker panels for early diagnostics of cardiotoxicity (148). Procollagen type I C-terminal propeptide (PICP), a direct product of collagen synthesis, is one of the most promising biomarkers for fibrosis. Research reveals that in breast cancer patients treated with anthracycline-based chemotherapy, a rise in serum PICP levels at 3 months post-chemotherapy is significantly correlated with concurrent subclinical left ventricular dysfunction as indicated by global longitudinal strain (149).

#### 5.1.3 Functional studies and clinical evaluation

The ECG stands as a foundational and prominent method for assessing chemotherapy-induced cardiotoxicity, due to its non-invasiveness, ease of use, and strong reproducibility (150). Normal ECG detects various changes because of cardiotoxicity like prolongation of the QT interval. It is a commonly identified marker of drug-induced arrhythmia. Several studies have found evidence of this effect. A QTc of more than 500 ms is considered hugely abnormal and has an increased risk of possibly fatal arrhythmias (151). Changes in the ST segment of an ECG can also indicate injury to the heart muscle (152). Nevertheless, traditional ECG has limited sensitivity towards subclinical injury of heart as much of the cardiotoxic changes may not be exhibited in the resting state, specifically, in the early stage.

Since numerous cardiotoxic events are dependent on the load, the dynamic monitoring and exercise stress assessment are essential to ascertain patients with hidden or atypical symptoms (153). Remote monitoring through portable single-lead or 12-lead ambulatory ECG devices can effectively capture paroxysmal arrhythmias and ischemic events. According to studies, a monitoring duration of 14–21 days using single-lead EGC after chemotherapy detects new-onset atrial fibrillation in 9% of patients and significant QTc prolongation in 6.6% of patients (151). This monitoring approach is essential for identifying asymptomatic or nonspecific symptomatic cases.

Meanwhile, the six-minute walk test is a submaximal exercise test. It is not equivalent to the exercise stress ECG or echocardiogram. The main value of the test is in determining patients' functional capacity and symptoms during simulation of daily activities. A prospective study showed that using capecitabine brought on cardiotoxicity in 16.7% of cases. Of these, over 80% (26/32) were induced during exercise stress testing with only a few occurring at rest (153).

### 5.2 Baseline risk assessment

The patient must undergo a cardiovascular risk assessment before starting cardiotoxic anticancer therapy (154). In 2022, the European Society of Cardiology (ESC) published the “Guidelines on Cardio-Oncology”, advising the use of the Heart Failure Association - International Cardiocancer Society (HFA-ICOS) risk stratification for standardized assessment (155). This tool classifies patients into four risk levels. It does so by comprehensively analyzing patient factors (such as age, traditional cardiovascular risk factors, and CAD), the type of conventional tumor treatment (cardiotoxicity of the treatment), and the interaction between the two. Depending on the patient's risk level, the suggested monitoring of the heart which is done before, during, and after the treatment of cancer is given information (Table 2) (156). Upon treatment initiation, the monitoring strategy should dynamically implemented according to the risk stratification. It is suggested for patients with high risk to recommend repeating echocardiograms (with attention to GLS changes) at pivotal treatment nodes (for example, every 2–3 chemotherapy cycles) and detect hs-cTn and BNP/NT-proBNP serially. It aids in recognizing functional decline before there is an alteration in LVEF. Patients taking medications with the potential to prolong the QT interval should receive regular ECG monitoring. If there is cardiovascular toxicity, it is suggested that the cardiology and oncology teams engage in a joint discussion regarding the risks and benefits of whether to continue or discontinue treatment. In the case of hypertension caused by vascular endothelial growth factor inhibitors, after blood pressure is controlled at a safe range by intensifying antihypertensive treatment, anti-cancer treatment can usually continue.

**Table 2:** Comprehensive management pathway for cardiovascular toxicity based on baseline risk stratification.

Risk stratification	During cancer treatment	First year after cancer treatment	Long term follow-up
Baseline cardiovascular toxicity risk assessment	Informing, advising, and supporting patients to promote a healthy lifestyle Management of CVRF and CVD		
Low risk patients	Standard surveillance	Assessment at 1 year after completion of cancer therapy	Annual CVRF assessment^1^ Reassessment if new cardiovascular signs/symptoms
Moderate risk patients risk patients	Cardiology referral	Assessment at 1 year after completion of cancer therapy	Annual CVRF assessment toxicity re-stratificationt 5 years^a^ Transthoracic echocardiography every 5 years
High and very high risk patients	Cardiology referral CVD prevention	Assessment at 3 months and 1 yearafter completion of cancer therapy	Annual CVRF assessment^a^ Transthoracic echocardiography at years 1, 3, 5 and very 5 years
	Cardiology referral if new cardiovascular signs/symptoms or CTR-CVT develop		

CVD, cardiovascular disease; CVRF, cardiovascular risk factors; CTR-CVT, cancer therapy-related cardiovascular toxicity, BP, blood pressure; ECG, electrocardiogram; HbA1c, glycated haemoglobin; NP, natriuretic peptides.

^a^ Annual cardiovascular risk assessment (including clinical review, BP, lipidprofile, HbA1c, ECG, and NP) and CVRF management is recommended in cancersurvivors who were treated with a potentially cardiotoxic cancer drug.

### 5.3 Diagnostic criteria

Accurate clinical diagnosis and grading of cardiotoxicity in gynecological cancer patients has its importance. The core lies in establishing a unified and standardized assessment framework to clearly define events, quantify severity, and guide subsequent treatment adjustments and long-term follow-up. When abnormalities are detected during monitoring or when patients present with related symptoms, a structured diagnostic process should be initiated. For a long time, there has been an absence of consistent definitions and diagnostic criteria for cancer treatment-related cardiotoxicity in clinical practice and research, which to some extent has led to non-standardized management and heterogeneity in outcome reporting.

To solve this problem, the recently released ESC “Cardio-Oncology Guidelines” provides an authoritative integrated framework, which includes clear consensus definitions for cancer treatment-related cardiomyopathy and heart failure (155). Meanwhile, the diagnostic criteria for other treatment-related cardiovascular events (including pericardial disease, valvular lesions, arrhythmias, etc.) are consistent with those for the general cardiac disease population. The guidelines recommend using “cancer treatment-related heart dysfunction"(CAR-RHD) to describe the reduction in cardiac systolic or diastolic function that occurs during or after cancer treatment (Table 3). This definition covers the continuous process from subclinical myocardial injury to symptomatic heart failure and recommends a comprehensive diagnosis based on symptoms, biomarkers, and imaging (especially GLS by echocardiography and CMR). This standardized framework can be integrated with the widely used Common Terminology Criteria for Adverse Events (CTCAE) (157)and heart failure classification of the American Heart Association (AHA)/American College of Cardiology (ACC) (158), thus providing the range from early detection, severity grading to long-term staging management, thus providing a clear and consistent basis for clinical decision-making and personalized intervention (Table 4).

**Table 3:** The definition of cancer therapy-related cardiac dysfunction in ESC guidelines on cardio-oncology.

CTRCD	Classification	Definition
Asymptomatic CTRCD	Severe	New LVEF reduction to < 40%
	Moderate	New LVEF reduction by ≥ 10 percentage points to an LVEF of 40–49% OR New LVEF reduction by < 10 percentage points to an LVEF of 40–49% AND either new relative decline in GLS by > 15%from baseline OR new rise in cardiac biomarkers
	Mild	LVEF≥50% AND new relative decline in GLS by > 15%from baseline AND/OR new rise in cardiac biomarkers
Symptomatic CTRCD (HF)	Very severe	HF requiring inotropic support, mechanical circulatory support, or consideration of transplantation
	Severe	HF hospitalization
	Moderate	Need for outpatient intensification of diuretic and HF therapy
	Mild	Mild HF symptoms, no intensification of therapy required

CTRCD, cancer therapy-related cardiac dysfunction; HF, heart failure; LVEF, left ventricular ejection fraction,.

GLS, global longitudinal strain.

**Table 4:** Integrated application framework of CTCAE, AHA/ACC staging and ESC guidelines.

Aspect	Common Terminology Criteria for Adverse Events (CTCAE)	AHA/ACC Heart Failure (HF) Stages	ESC Guidelines on Cardio-Oncology
Core Purpose	A standardized grading system for adverse events in oncology trials and practice.	A conceptual disease-progression model emphasizing prevention, for all HF aetiologies.	A clinical practice guideline with an inclusive risk assessment, monitoring, and management pathway.
Primary Function	To offer a unified terminology which can be used to describe and grade severity of adverse events.	The objective is to classify patients in the course of HF development for risk stratification.	To present actionable clinical algorithms for the prevention, early detection, and management of cardiovascular toxicities.
Classification	Grades 1–5 for each adverse event term. • Grade 1: Mild; asymptomatic or mild symptoms. • Grade 2: Moderate; minimal intervention indicated. • Grade 3: Severe or medically significant; hospitalization indicated. • Grade 4: Life-threatening consequences; urgent intervention indicated. • Grade 5: Death related to adverse event.	Stages A-D • Stage A: At risk for HF, but no structural heart disease or symptoms. • Stage B: Structural heart disease without signs/symptoms of HF. • Stage C: Structural heart disease with current or prior HF symptoms. • Stage D: Refractory HF requiring specialized interventions.	• Risk Stratification and Management Pathways: • Pre-treatment risk stratification (e.g., using the HFA-ICOS risk tools). • Structured monitoring protocols based on risk and therapy. • Clear definitions (e.g., for cancer-therapy-related cardiac dysfunction). • Treatment for specific toxicities.
Key Limitations	A descriptive grading system. It focuses on event severity but does not provide management pathways.	A conceptual staging framework. It does not specify exact monitoring frequencies or treatment protocols.	A detailed guideline. Its implementation requires clinical resources and MDT coordination, and recommendations may evolve with new evidence.

CTCAE, common terminology criteria for adverse events; AHA, American Heart Association; ACC, American College of ESC, European Society of Cardiology; HF, heart failure.

## 6 Strategies for the prevention and management of cardiovascular toxicity

### 6.1 Optimization and selection of treatment plans

It is necessary to optimize treatment regimens to prevent cardiovascular toxicity. The main principle is to select the less cardiotoxic from available treatment options with equal antitumor efficacy and perform individual adjustments in high-risk patients (159). Prior to developing a treatment regimen, it is important to evaluate the patient's baseline cardiovascular status, traditional risk factors (e.g., hypertension, diabetes), and tumor attributes. For instance, proper control of baseline blood pressure in patients who are supposed to get vascular endothelial growth factor inhibitor therapy significantly lowers the frequency and intensity of treatment-related hypertension (160). Studies show that patients with advanced tumors are at an increased risk of cardiac dysfunction after anthracyclines or anti-HER2 targeted therapies (131). When selecting a drug, liposomal formulations (e.g., liposomal doxorubicin) can be a substitute for standard anthracyclines as they alter the pharmacokinetic distribution to prevent cardiotoxicity. In addition, precision medicine technologies such as the liquid biopsy and molecular profiling can refine the choice of targeted therapies based on tumor genetics, which avoids exposure to poor therapeutic and cardiotoxic chemotherapeutic agents.

### 6.2 Application of cardioprotective agents for prophylaxis

The use of cardioprotective agents for prophylaxis is a key strategy to reduce cardiovascular toxicity in high-risk patients. Currently, dexrazoxane is the only medication approved in the United States Food and Drug Administration for preventing damage to the heart from anthracyclines. The substance works by chelating iron ions, preventing the formation of harmful complexes and harmful oxidative damage. Patients receiving a high cumulative dose of anthracyclines or presenting multiple risk factors should receive this agent (21). Furthermore, drugs such as angiotensin-converting enzyme inhibitors or *β*-blockers have been studied to reduce heart dysfunction related to chemotherapy (160). Furthermore, phosphodiesterase-5 inhibitors have shown promising cardioprotective effects in preclinical studies (161, 162).

Certain natural products, such as Withaferin A, show strong anti-ovarian cancer activity. They also offer cardioprotective ability and can thus contribute to drug discovery efforts in the future (162).

Recent innovations in strategies to protect the heart from the toxic effects of cancer treatment look promising. Mitochondrial transplantation as a new experimental proposal has presented. According to preclinical studies, exogenous healthy mitochondria therapy can ameliorate myocardial energy metabolism and even reverse doxorubicin-induced cardiac systolic dysfunction, providing a new insight for intervention of chemotherapeutic agent–induced cardiomyopathy (163). In terms of drug translation, sodium-glucose cotransporter 2 (SGLT2) inhibitors like dapagliflozin and empagliflozin induce cardioprotective effects beyond glycemic control by enhancing myocardial energy metabolism, reducing oxidative stress, and alleviating fibrosis (164, 165). At the same time, the active components of traditional Chinese medicine such as Danshen and ginsenosides have shown cardioprotective capacity in preclinical studies through their properties of antioxidants and anti-inflammatories (166, 167). Most of the data originates from animal studies as well as small observations. So, it faces issues like difficult composition, unknown mechanism of action, and quality control problems. Thus, it is essential to conduct large-scale clinical trials to confirm their safety and efficacy in modern-day clinical practise.

### 6.3 Monitoring and intervention during treatment

The key to balancing anticancer effectiveness and cardiovascular safety is dynamic monitoring. Detection of subclinical injuries may benefit from a combination of cardiac imaging studies and serum biomarkers such as troponin and natriuretic peptides (145). Due to the intricate nature of the underlying cause, the need to create and treat will require a multidisciplinary team (MDT) of gynecologic oncologists, cardiologists, imaging specialists and clinical pharmacists. As soon as an early sign of cardiotoxicity is recognized, multidisciplinary evaluation should be started immediately so that management can be individualized. The evaluation of the side effects of anticancer treatment requires a holistic approach that takes into account the severity of the symptoms, the stage of the disease, the efficacy of treatment, the overall health status of the patient as well as the balance between benefit from ongoing anticancer treatment and risk of CAD. Management has three key goals: preventing toxicity, maintaining effective antitumor treatment, and preserving long-term cardiovascular health. Based on the type of toxicity, strategies should be designed accordingly.

For secondary hypertension due to antiangiogenic treatment, target blood pressure must be less than 140/90 mmHg. For patients with diabetes or renal failure, the recommended target is stricter, it is <130/80 mmHg. Angiotensin-converting enzyme inhibitors (ACEIs) and angiotensin II receptor blockers (ARBs) are first-line agents that spare endothelial function and lack impairment of antitumoral efficacy. If monotherapy shows insufficient response, a combination with long-acting calcium channel blockers may be useful (160). In cases of left ventricular systolic dysfunction (reduced LVEF), management should adhere to established heart failure guidelines. The key medications includes ACEIs/ARBs, *β*-blockers and aldosterone antagonist (168).

In case of arrhythmias, one must first assess the severity of the problem, presence of symptoms, effect on hemodynamics, and proactively search and fix the precipitants. For instance, inotropic drug levels, myocardial ischemia, heart failure, infections, and electrolyte imbalance should be managed. Patients on anti-angiogenic therapy or any additional thrombotic risk factor should be monitored for VTE (169). Treatment decisions must balance the benefits of anticoagulation against potential bleeding risks. For high-risk patients, prophylactic anticoagulation therapy may be considered, but a comprehensive assessment of bleeding risk is necessary. In the event of VTE, appropriate anticoagulants should be selected in accordance with guidelines and closely monitored, such as low molecular weight heparin, vitamin K antagonists, or novel oral anticoagulants.

### 6.4 Long-term follow-up and survivor care

Due to improved survival in cancer patients the late cardiac adverse effects of the anticancer therapy have now become a major concern for long-term survival and quality of life (170). Individualized long-term cardiac follow-up plans should be developed for patients who have received potentially cardiotoxic treatment such as high-dose anthracyclines or anti-HER2 targeted therapy. Annual or two-yearly cardiac imaging and biomarker measurement should form part of these plans. Patients should be followed up on a regular basis with periodic assessment of cardiovascular risk factors, clinical symptoms, cardiac biomarkers (e.g., BNP/NT-proBNP), and imaging (e.g., echocardiography) even if cardiac function is normal at the end of treatment. In addition, Cardiovascular complications have a multi-dimensional impact on quality of life: on the physiological level, it is manifested as decreased cardiac function and physical symptoms caused by arrhythmia. If LVEF is reduced by five percent, there is a 40% risk of restricted daily activities. On the psychological level, it is mainly reflected in the increased incidence of depression and anxiety. Research data shows that the proportion of diagnosed depression among cancer survivors with concomitant cardiovascular disease is 38.7%, significantly higher than that of patients with a single disease (171). This interaction between the body and mind forms a vicious cycle, exacerbating the deterioration of quality of life. Therefore, the long-term management of cancer survivors should establish an integrated follow-up model, with cardiovascular health and quality of life assessment as core components, and through comprehensive measures such as cardiac rehabilitation, psychological support, and social intervention, comprehensively improve the long-term well-being of patients (172). At the same time, it is especially important to actively manage the classic cardiovascular risk factors like hypertension, hyperlipidaemia, diabetes and obesity, and promote a healthy lifestyle through quitting smoking, regular workouts and balanced diet (173).

## 7 Discussion

While therapeutic advances for gynecological cancers have markedly improved patient survival, they have concurrently elevated cardiovascular toxicity to a central determinant of long-term quality of life. This review systematically delineates the cardiovascular risk profiles, mechanistic underpinnings, and management strategies associated with standard anti-cancer treatments for gynecological malignancies. Consequently, we posit that a comprehensive evaluation and proactive management of cardiovascular toxicities, carefully tailored to the specific disease context and patient profiles in gynecological oncology, are imperative for optimizing long-term clinical outcomes.

Gynecological cancers encompass various types such as cervical, ovarian, and endometrial cancers, affecting a wide age range from young women to postmenopausal elderly patients. Cardiovascular risk management must therefore be adapted to different life stages and underlying health conditions. Endometrial cancer patients often present with metabolic syndrome manifestations like obesity and diabetes, creating a high-risk background that predisposes them to hypertension when receiving treatments such as VEGF inhibitors. On the other hand, younger patients, including those with cervical cancer and ovarian cancer, may be treated over a range of decades with possible longer-term cardiovascular impacts despite having lower baseline cardiovascular risk. In addition, modern multimodal, sequential comprehensive treatments (surgery, chemotherapy, targeted maintenance, immunotherapy) are exposing the cardiovascular system to repeated insults from different mechanisms, making cumulative risk non-negligible. The long-term metabolic and vascular effects of endocrine therapies commonly used in gynecology also need to be incorporated into holistic management. Thus, the three dimensions of cancer type, individual characteristics and treatment regimen must be integrated into cardiovascular risk assessments.

With the advancement of treatment options, the focus of cardiovascular toxicity concerns has shifted significantly. Through formulation optimization such as PLD and rational restrictions on clinical use, the dose-dependent myocardial injury risk from conventional anthracyclines has been effectively controlled. Today's targeted therapies (anti-angiogenic agents, PARP inhibitors) have shifted the main toxicities to hypertension, thrombosis, and metabolic disorders.

Their core mechanisms are closely related to vascular endothelial dysfunction and activation of the RAAS. This calls for a shift forward in clinical management with an increased focus on blood pressure monitoring and metabolic regulation. Immune checkpoint inhibitors pose a more advanced challenge. While the occurrence of immune-related myocarditis caused by them is rare, its fatality is high and the mechanism differs. This phenomenon is indicative of the expansion of the cardiovascular toxicity spectrum to a new dimension called immune-mediated injury. Therefore, the clinical response strategies must be adapted to address the new scenario of co-existing multiple mechanisms.

There has been a shift from management by reactive means to more proactive prevention and management for cardiovascular toxicity. Current the ESC guidelines currently stress the importance of monitoring assessment. A MDT which comprises the gynecologic oncologist, cardiologist, clinical pharmacist, etc. should be involved in treatment decision making. Indicators of subclinical myocardial injury damage include GLS and hs-cTnT/I, which should gradually enter routine monitoring to improve the risk warning window. Patients requiring cardiotoxic regimens that cannot be avoided should be cardioprotected. Interventions may include prophylactic dexrazoxane administration for patients receiving anthracyclines, optimization of antihypertensives for patients receiving anti-angiogenic therapy and supplementation with lifestyle interventions such as exercise rehabilitation.

Nonetheless, there are significant research gaps in this field: long-term data regarding cardiovascular outcomes from large cohorts of gynecological cancer patients are lacking. The assessment of cardiovascular safety of new agents such as PARP inhibitors and antibody-drug conjugates is still in its infancy. Therefore, there is an urgent need for prospective clinical research and translational medicine exploration targeting gynecological cancer populations. This will help build more accurate risk prediction models, validate specific protective interventions, and ultimately achieve a balance between oncological efficacy and long-term cardiovascular health benefits.

## 8 Conclusion

In summary, the management of cardiovascular toxicity associated with gynecological anti-cancer therapy is a dynamically evolving and increasingly important interdisciplinary field. Clinicians must fully recognize the uniqueness of the patient population, the rapid evolution of treatment modalities, and the consequently changing toxicity spectrum. By integrating multidisciplinary resources and implementing risk assessment-based strategies for prevention, early monitoring, and proactive intervention, we can effectively control the cancer while maximizing the protection of patients' cardiovascular health, ultimately achieving the therapeutic goal of simultaneously improving both survival time and quality of life.
